# Evaluation of Soil Contamination Indices in a Mining Area of Jiangxi, China

**DOI:** 10.1371/journal.pone.0112917

**Published:** 2014-11-14

**Authors:** Jin Wu, Yanguo Teng, Sijin Lu, Yeyao Wang, Xudong Jiao

**Affiliations:** 1 College of Water Science, Beijing Normal University, Beijing, China; 2 China National Environmental Monitoring Center, Beijing, China; University of Kansas, United States of America

## Abstract

There is currently a wide variety of methods used to evaluate soil contamination. We present a discussion of the advantages and limitations of different soil contamination assessment methods. In this study, we analyzed seven trace elements (As, Cd, Cr, Cu, Hg, Pb, and Zn) that are indicators of soil contamination in Dexing, a city in China that is famous for its vast nonferrous mineral resources in China, using enrichment factor (EF), geoaccumulation index (I_geo_), pollution index (PI), and principal component analysis (PCA). The three contamination indices and PCA were then mapped to understand the status and trends of soil contamination in this region. The entire study area is strongly enriched in Cd, Cu, Pb, and Zn, especially in areas near mine sites. As and Hg were also present in high concentrations in urban areas. Results indicated that Cr in this area originated from both anthropogenic and natural sources. PCA combined with Geographic Information System (GIS) was successfully used to discriminate between natural and anthropogenic trace metals.

## Introduction

Environmental issues that pose a threat to soil health include erosion, a decline in organic matter content and biodiversity, contamination, sealing, compaction, salinization, and landslides [1]. In China, contamination is recognized as a major threat to soil. In recent years, there have been numerous review and research articles providing assessments of various kinds of soil contamination, including urban soil contamination, agricultural soil contamination, and soil contamination in mining areas [2]. Several studies have also provided a comparison of the results of different methods for the assessment of soil contamination [3]–[5]. Such studies help to raise public awareness of soil contamination and to facilitate research on contamination and contamination control strategies. However, the status and trends of soil contamination, especially at regional scales, have not been well described. Knowledge of soil geochemistry is fundamental to assessing soil contamination at the regional scale. One of the most efficient tools for studying environmental geochemistry problems is geographical information system (GIS) based on geostatistical analysis. To our knowledge, maps and comparisons of indices derived from different soil contamination methods are not widely available.

The objective of our work was to determine the origin of trace metals in soils using various indices based on geochemistry mapping, including enrichment factor (EF), geoaccumulation index (I_geo_), and pollution index (PI), along with principal component analysis (PCA); we also aimed to critically evaluate the advantages and limitations of these methods. The data we used were obtained from a regional geochemical survey carried out in Dexing, a city in China that is famous for its vast nonferrous mineral resources. To better understand the outcome of this work, we first present a brief overview of core issues and problems associated with current soil contamination assessment methods.

### Selection of reference values

A major methodological problem associated with correctly assessing soil contamination is the identification of appropriate reference values for uncontaminated soil conditions, since all quantitative assessment methods rely on reference values of background concentrations [6]. The background, the crust, and the regulatory reference values are common reference values used for soil contamination assessment; the background value is the most appropriate reference value to evaluate soil contamination for theoretical considerations alone.

There is some variability in the definition of background. A selection of definitions and relevant terms is presented in Table 1 [7], [8], [9]. Indiscriminate usage of the term “background” to evaluate soil contamination can result in misinterpretations if several flaws are ignored. Reimann and de Caritat critically discuss the definitions and use of background values in environmental geochemistry [10]. Some characteristics are summarized:

**Table 1:** A selection of definitions of background and relevant term.

Definition	Term	Reference
The normal abundance of an element in barren earth material,and it is more realistic to view background as a range ratherthan an absolute value	Background	[7]
Geogeneous or pedogeneous average concentration of asubstance in an examined soil	Background	[8]
If the atmosphere in a particular area is polluted by somesubstance from a particular local source, then the backgroundlevel of pollution is that concentration, which would existwithout the local source being present.	Background	[9]
Widely used to infer background levels reflecting naturalprocesses uninfluenced by human activities.	Natural background	[10]
used to describe the unmeasurably perturbed and no longerpristine natural background	Ambient background	[10]
Used when data either come from age-dated materials or arecollected from areas believed to represent a survey/studyarea in its supposed preindustrialization state.	Pre-industrial background	[10]
The outer limit of background variation	Threshold	[11]
A depature from the geochemical patterns that are normal fora given area or geochemical landscape	Anomaly	[7]
Concentrations of substances characterizing variability in thegeochemistry of earth’s surface materials and are needed fordocumenting the present state of the surface environmentand to provide datum against which any changes canbe measured	Baseline	[12]

1. No specific global background levels of elements can be defined. Natural element concentrations can be as high or even higher than any visible anthropogenic contamination, therefore it is difficult to identify anthropogenic additions and contamination in most cases.
2. Background levels depend on location and scale, and should usually be restricted to the local scale. It has been demonstrated that background levels may vary both within and between regions.
3. It is more realistic to view background as a range rather than an absolute value. There are a range of values characterizing any particular area or region that reflect the heterogeneity of the environment.
4. It can be argued that natural background no longer exists on this planet. There is evidence from the world’s ice sheets and glaciers that small amounts of elements have been transported on intercontinental scales to remote regions and deposited as a result of being released into the atmosphere from human activities.

Threshold is usually expressed as a single value showing the upper background between anomalous and background concentrations, while the baseline, usually expressed as an observed or 95% expected range, is used mainly in geochemical exploration, and is not appropriate for environmental purposes. The background values derived from different percentiles of trace metal soil concentrations for some countries are summarized in Table 2 [11]–[16]. The use of percentile as an upper background (threshold) provides a practical approach to continue to use the term “background”. This implies the availability of reliable procedures to evaluate soil contamination, but raises the question of data comparability.

**Table 2:** Summary of often used background values of trace metal soil concentrations (mg/kg).

Country	As	Cd	Cr	Cu	Hg	Ni	Pb	Zn
Austria[13]	nd	0.37	50	35	0.19	40	28	111
China[14]	11.2	0.1	61	22.6	0.07	26.9	26	74.2
Estonia[13]	nd	0.52	30	24	0.08	29	21	58
Germany[13]	nd	1.50	45	45	0.45	38	171	225
Japen[14]	9.02	0.41	41.3	36.97	0.28	28.5	20.4	63.8
Jiangxi (China)[15]	10.4	0.10	48.0	20.8	0.08	19	32.1	69.0
Lithuania[13]	nd	nd	23	13	nd	19	15	37
Medium of world[16]	5	0.3	80	25	0.05	20	17	70
Netherlands[13]	nd	0.60	74	27	0.23	38	42	110
Romania[13]	nd	1.23	37	35	nd	50	38	167
Slovakia[13]	Nd	0.33	55	38	0.14	41	23	85
The Continental Crust[16]	1.7	0.1	126	25	0.04	56	14.8	65
United States[14]	7.2	nd	54	25	0.09	19	19	60
United Kingdom[14]	11.3	0.62	84	25.8	0.1	33.7	29.2	59.8
Upper continental crust[16]	2	0.1	35	14.3	0.06	18.6	17	52
Interval	1.7–11.3	0.1–1.5	23–126	13–45	0.04–0.45	18.6–56	14.8–171	37–225
Range	9.6	1.4	103	32	0.41	37.4	156.2	188
Relative range	85%	93%	82%	71%	91%	67%	91%	84%

When local information is unavailable, and more cannot be obtained, it is necessary to resort to data generated by surveys from different parts of the world covering spatially significant areas (Table 2). The average concentrations of 90 naturally occurring elements in the Earth’s crust have been estimated; these are known as “Clarke values” and can be found in Taylor and Wedepohl [17], [18]. These two papers summarize published data on the composition of the upper continental crust, which varies slightly because there are hypothetical concentrations based on assumed proportions of various crustal rock types. The concentrations of elements differ so widely from one geologic unit to another, that the use of the Clarke value for an element in a regional or local context does not sufficiently represent variations in element distributions caused by mineralization or contamination in a particular sampling medium [19]. However, such values can give a preliminary indication of whether results from a new investigation are within an expected range and whether they reflect natural variations in concentrations present in different environments [10].

The use of regulatory reference value (RRVs), which are generally based on background values in combination with toxicity levels, is a different approach to evaluating soil contamination. RRV is set by a state authority, and is not always based solely on scientific evidence, but also on economic or political considerations. The RRVs for trace metals in soil of some countries are provided in Table 3 [20]–[22]. RRVs have been given various names in their original languages that translate in English to maximum admissible concentration values, target values, intervention values, guideline, cut-off values, and many others. Advantages of using screening values have been pointed out by several authors [23], [24] and are confirmed in practice by their long term and successful use in many countries. Advantages include their speed and ease of application, their clarity for use by regulators and other non-specialist stakeholders, and their comparability and transparency. The major limitation of screening values is that crucial site-specific considerations cannot be included. Screening values may give rise to a misleading feeling of certainty, knowledge, and confidence, which can lead to reluctance on the part of users to apply them to site-specific risk assessments [25]. A combined approach, using guideline values to streamline the preliminary stages of decision making and site-specific risk assessment to achieve fine-tuning in later stages of an investigation, is generally considered the most appropriate [26].

**Table 3:** Summary of regulatory reference values of trace metal in soil of some countries (mg/kg).

Countries	Denomination	As	Cd	Cr	Cu	Hg	Ni	Pb	Zn
Austria[6]	Guidelines		0.5–1	100	100	1	60	100	300
Chinese[20]	Guidelines	30	0.3	200	100	0.5	50	300	250
Canada[21]	Residential/parkland guidelines	12	10	64	63	6.6	50	140	200
Canada[21]	Commercial guidelines	12	22	87	91	24	50	260	360
Canada[21]	Industrial guidelines	12	22	87	91	50	50	600	360
Germany[6]	Clay		1.5	100	60	1	70	100	200
	Loam/silt		1	60	40	0.5	50	70	150
	Sand		0.4	30	20	0.1	15	40	60
The Netherlands[22]	Target guidelines	29	0.8	100	36	0.3	35	85	140
The Netherlands[22]	Intervention guidelines	55	12	380	190	10	210	530	720
Switzerland[6]	Guidelines		0.8	50	40	0.5	50	50	150

Based on the location of a reference area in relation to a study site, two types of reference areas can be classified: on-site and off-site. All the statistically derived references mentioned above are off-site references and are easy to compute. Desaules argued that off-site reference methods are obviously not appropriate to assess weakly contaminated sites, while the specific and sensitive on-site reference method could be used to accurately identify soil contamination based on the observed values of investigated trace metals [6]. On-site reference is a value specific to a particular material and to a particular locality.

Deep soil layer values are not affected by contamination and are considered to be the most convenient for use as on-site references of the same soil profile [27]. There is debate about the use of deep soil layer values to evaluate soil contamination. The use of deep soil layers, instead of the continental crust, as a reference value improves the sensitivity of EF to anthropogenic surface enrichments [27], [28]. In contrast to other authors who have promoted the use of deep soil layer values, Reimann and de Caritat demonstrate that it does not significantly reduce the shortcomings of the EF approach and may even give spurious results based on results from subcontinental-scale geochemical surveys [10].

Other suggestions for on-site references to identifying contamination are buried fossil topsoils, provided the buried soils have not been contaminated or depleted subsequently by pedogenic processes, and dated peat bog samples, which make it possible to trace the chronology of atmospheric deposition [6], [29], [30]. However, both these types of bog samples are difficult to obtain.

### Indices and methods for the assessment of soil contamination

Popular soil contamination assessment methods can be classified into two categories: quantitative and qualitative. The qualitative methods, such as PCA, factor analysis, and cluster analysis, are inferential and indicative. These multivariate analyses require that each variable shows a normal distribution and that the whole dataset shows a multivariate normal distribution [31]. Some of the most commonly used quantitative methods are the contamination factor (CF), enrichment factor (EF), and geoaccumulation index (I_geo_). The CF, defined by Hakanson, enables an assessment of soil contamination through the use of concentrations in the surface layer of bottom sediments to preindustrial levels as a reference [32]. In China, the CF was adopted as a pollution index (PI), which is often evaluated by comparing metal concentrations with related environmental guidelines, or with respect to relevant background values. The CF is sometimes used in equivalency to background. The PI will be used in this paper because it has been widely used in soil contamination assessments. EF was introduced in the 1970s, and was initially developed to obtain information on the origin of elements in the atmosphere [33], [34]. I_geo_, a method used for the evaluation of the degree of contamination in aquatic sediments was originally defined by Müller and has been widely used in soil trace metal studies [35]. There are numerous studies which use the abovementioned factors to assess soil contamination at different scales [36], [37], while, several studies use a combination of methods [38]–[40].

Care needs to be taken when using the terms ‘contamination’ and ‘pollution’. Contamination is the presence of a substance where it should not be, or in levels that are above background levels [29]. The term pollution is defined as contamination that results in adverse biological effects [29]. In the context of soil systems, the difference between contamination and pollution is that contamination is presence of the substance in soil adversely affecting the soil, and pollution is the presence of the substance in the soil adversely affecting the usefulness of the soil [41]. The sources of trace metals in soils are manifold, and include natural parent materials and various exogenous pollution sources [42]. Identifying and quantifying anthropogenic trace metals in soil is crucial for the assessment of soil contamination. However, difficulties arise from correctly evaluating the degree of soil contamination, especially at slightly disturbingly area. Generally, local hotspots of soil contamination (such as metal smelters and brownfields) are easier to identify and delimitate than regional contamination by agrochemicals and atmospheric deposition close to urban or industrial sources, or global contamination by long-range transboundary air contamination [6]. There is no soil contamination assessment method available to provide accurate information on the extent of perturbation for a number of reasons.

The formation of soil is a function of climate, soil organisms, landscape, plants, time, and geology. All of these factors can affect the concentration of any one element in a soil system. Because different sample materials will respond differently to the input of an element, it is not appropriate to use a single value (e.g., mean, maximum) to evaluate soil contamination of an entire area. There are two methods to describe characteristics of contamination over an entire area: the calculation of the proportion of contaminated samples in a given area, and geochemical mapping. However, the proportion of contaminated samples does not represent the specific geochemical context of each sample or other relevant information, so that the proportion calculated will not reliably provide a complete picture of soil contamination of a given area. Geochemical mapping, usually performed on GIS, provides a visual representation of the geochemical and contamination processes related to the distribution of trace elements. Additionally, most current soil contamination assessment frameworks are limited to potentially toxic inorganic trace metals (As, Cd, Cr, Cu, Hg, Mn, Pb, and Zn); it is important to also consider other important inorganic (F, P, and Se) and organic (PAHs, PCBs, and PCDD/Fs) substances.

## Materials and Methods

### Study site description

**Figure 1:**
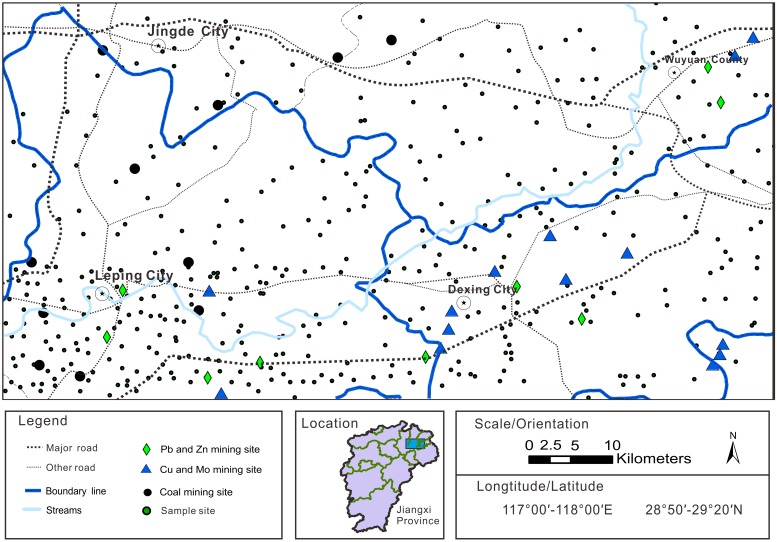
Location of the study area and sampling pattern.

The study area is located in the northeast part of Jiangxi province (117°00′−118°00′E, 28°50′−29°20′N), China (Figure 1). No specific permissions were required for these locations. The field studies did not involve endangered or protected species. The altitude ranges from 20–1300 m, and the climate zone is subtropical monsoon, with an annual average temperature of 17°C and rainfall of 1900 mm. The soils are mainly classified as paddy soil in the plains, and yellow soil and red soil in the hilly areas. The stratum is full-fledge and spread across the study area, except for areas containing Silurian, Devonian, and Tertiary strata [43]. The Lean River is the main water body in the study area and has a number of branches, including the Jishui River, the Dawu River, and the Changle River.

### Sampling and analyzing

From December 2003 to April 2004, 407 non-agricultural topsoil samples (0–20 cm) were collected using a Global Positioning System (GPS) to identify sampling locations (Figure 1). The area covered by the sampling sites was approximately 400 km^2^. One sample per 16 km^2^ was collected at sites far from potential contamination sources, and one sample per 4 km^2^ was collected around potential contamination sources, such as the Dexing copper mine, and the Leping coal mine. Each sample represents composite material taken from four points over a 1-km^2^ patch of land; total sample weight was 1–1.5 kg. Samples were air dried at 35–40°C prior to analysis. The soil was passed through a 6-mm sieve to remove stones and plant material, then was milled with a carnelian mortar then passed through a 0.015-mm sieve prior to chemical analysis.

Each soil sample (10–20 mg) was digested in 1 mL of 60% (w/w) HNO_3_ and 1 mL of 60% (w/w) HClO_4_ in a stainless steel high-pressure digestion bomb at 140°C for 6 h. After completely cooling the system, the open vial was transferred to a hot plate (about 190°C) to evaporate the solution until the volume had decreased to several hundred micro-liters, then 0.5 mL of 49.5% (w/w) HF was added and the sample was evaporated again. The HF treatment was repeated several times until the silicate minerals had been completely dissolved. Finally, the residual solution was diluted to 6 mL with 1% (w/w) HNO_3_, filtered through a syringe filter (0.45 µm). Total concentrations of Cu, Pb, Zn, and Cr were analyzed by inductively coupled plasma atomic emission spectroscopy, As and Hg were analyzed by atomic fluorescence spectroscopy, and Cd was analyzed by atomic absorption spectroscopy. The total concentrations of K, Ca, Na, Mg, Si, Al, Mn, Ti, and Fe were determined by wavelength-dispersive X-ray fluorescence spectroscopy. Quality assurance and quality control procedures were performed along with laboratory analyses through the analysis of standard reference materials GSS-1, GSS-2, GSS-3, and GSS-4 soil (National Research Center for Geoanalysis of China). The results showed that the precision and bias of the analysis were generally below 5%. Recoveries of samples spiked with standards ranged from 95 to 105%.

### Soil contamination assessment method

The assessment of soil contamination was carried out using EFs, I_geo_, and PIs. To enable a comparison of the three indices, the value of the EFs, I_geo_, and PIs were calculated using the modified formula based on the equations suggested by Chester and Stoner, Hakanson, and Müller, respectively [32], [33], [35].
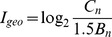
(1)

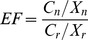
(2)

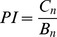
(3)where *C_n_* is the concentration of the element in the soil environment, *B_n_* is the background concentration of soil in Jiangxi, *X_n_* is the concentration of the reference element in the soil environment, and *X_r_* is the concentration of the reference element in the reference environment. For this study, we used Al_2_O_3_ as the reference element.

For comparison of the degree of contamination, soil contamination indices were divided into five grades according to their classification criteria (Table 4). The classification for I_geo_ and PI were adjusted based on the definitions given by Müller and Hakanson and the classification of EF was done according to Sutherland [32], [35], [44].

**Table 4:** Classification of different soil contamination assessment models.

Index class	I_geo_	EF	PI	Description of classes
**1**	I_geo_<0	EF<2	PI<1	Uncontaminated
**2**	0≤I_geo_<1	2≤EF<5	1≤PI<3	Moderately contaminated
**3**	1≤I_geo_<3	5≤EF<20	3≤PI<6	Considerable contaminated
**4**	3≤I_geo_<5	20≤EF<40	6≤PI<12	High contaminated
**5**	5≤I_geo_	40<EF	12<PI	Extremely contaminatied

### Multivariate statistics analysis

In this study, principal component analysis was conducted to identify the relationship between heavy metals in soil and their potential sources. The common two potential sources were: natural (the biogeochemical processes of parent material and the physicochemical processes of parent material) and anthropogenic (industrial activity, industrial activity, vehicle-related activity and fossil energy activity). PCA is designed to reduce a dataset containing a large number of variables to a smaller size by finding a new set of variables called components. Is this study, there are 10 element measurements constituting the variables, and hence 10 components. PCA was conducted using a commercial statistics software package SPSS (version 17) for Windows. The assumption of normality for all variables was checked before multivariate statistical and spatial analyses; when necessary, data transformation was done via a Box-Cox transformation.

### Geostatistical Analysis

The Kolmogorov–Smirnovtest (p<0.05) indicated that the various metals had skewed concentration distributions. Only As and Zn fitted a normal distribution after being logarithmically transformed. A log transformation was conducted prior to the analysis because of the skewed distributions of the heavy metal data.

Ordinary kriging is the most commonly used interpolation method to predict the overall trend of soil pollution. However, for the purpose of identifying contaminated areas, inverse distance weighting (IDW) is more appropriate to predict local features of soil pollution, especially local hotspots and cold spots [45]. It is a deterministic spatial interpolation model that is directly related to the values being estimated, and is suited to small datasets for which modeled semi-variograms are very difficult to fit. The interpolating function is:
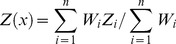
(4)

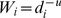
(5)Where Z(x) is the predicted value at an interpolated point, W_i_ is the weight assigned to point i, Z_i_ is at a known point, d_i_ is the distance between point i and the prediction point, and n is the number of known points used in the interpolation. Interpolation mapping was conducted using IDW within ArcGIS 9.30 software.

## Results and Discussion

### Descriptive statistical analysis

Descriptive statistics of heavy metal concentrations of topsoil are presented in Table 5. The arithmetic means concentrations of As, Cd, Cr, Cu, Hg, Pb, and Zn were 11.63, 0.24, 72.09, 53.48, 0.10, 47.02, and 87.98 mg/kg, respectively. Wide concentrations ranges coupled with the relatively high CV values for metal elements demonstrate the anthropogenic contribution in the study area. In this study, the Coefficient of variance was higher for Cu than for the other metals, and their concentrations varied widely. This suggests that Cu inputs to the soil in the study area may be attributable to anthropogenic sources.

**Table 5:** Descriptive statistics of metal levels (mg/kg) and selected properties (%) in soil.

Parameter	Range	Mean	S.D.	C.V.(%)	Skewness	Kurtosis	Local background value[46]
**As**	1.80–52.10	11.63	7.15	0.61	2.05	5.78	19.00
**Cd**	0.04–1.55	0.24	0.19	0.79	3.55	16.21	0.17
**Cr**	9.90–659.00	72.09	33.92	0.47	12.87	221.56	92.00
**Cu**	5.60–629.00	53.48	69.25	1.29	4.13	20.75	48.00
**Hg**	0.03–0.80	0.10	0.06	0.60	7.68	77.99	0.15
**Mn**	88.00–916.00	307.28	159.54	0.52	1.51	2.33	854.00
**Pb**	16.00–244.00	47.02	29.84	0.63	3.85	17.82	47.00
**Ti**	1082.00–9555.00	5429.92	922.53	0.17	−0.76	5.11	6320.00
**Zn**	27.20–799.00	87.98	55.75	0.63	7.30	76.34	108.00
**Al_2_O_3_**	7.30–20.65	13.51	2.05	0.15	−0.15	0.26	19.08
**Fe_2_O_3_**	1.57–9.64	4.31	1.11	0.26	0.91	2.36	6.44
**K_2_O**	0.77–4.80	2.25	0.63	0.28	0.31	1.31	3.06
**MgO**	0.22–3.64	0.65	0.29	0.45	3.76	31.04	0.90
**Na_2_O**	0.06–3.03	0.37	0.25	0.68	3.79	31.18	0.55
**SiO_2_**	35.93–83.41	69.47	4.97	0.07	−0.71	4.94	74.37

The mean concentrations of all metals, especially Cd and Cu, exceeded the environmental background values for Jiangxi and China [14]. This was probably because of the influence of mining activities in the study area. It was found that the highest concentrations of all heavy metals were higher than their corresponding guidelines for soils, except Pb, based on the Chinese Environmental Quality Standard for Soils [20]. However, the mean concentrations of the metals were lower than the guidelines. The mean concentrations of most metals, except Cd, Cu and Pb, were lower than the background values of local.

### Soil contamination assessment based on EF

The descriptive statistics of EF corresponding to the seven trace elements measured in the study area are given in Table 6. Mean values of EF were less than 2 for As and Zn, indicating no contamination by those metals in the soil. The mean EF values of Cd, Cr, Cu, and Pb ranged from 2.09 to 3.58; with respect to those metals, the soil was classified as moderately contaminated. The mean EF value of Hg was approximately 20, which was the highest of all the metals and which indicates considerable soil contamination.

**Table 6:** Descriptive statistics of soil contamination indices for soil trace metal.

Variable	Min	Max	Mean	Std	CV (%)	skewness	kurtosis
**EF(As)**	0.23	8.69	1.60	0.99	61.88	2.32	8.86
**EF(Cd)**	0.72	25.45	3.54	2.78	78.53	3.73	18.85
**EF(Cr)**	0.26	16.25	2.14	0.87	40.65	10.55	167.87
**EF(Cu)**	0.32	38.34	3.58	4.36	121.79	3.98	19.06
**EF(Hg)**	0.59	13.58	18.30	1.12	6.12	5.59	47.21
**EF(Pb)**	0.93	11.10	2.09	1.33	63.64	3.98	19.30
**EF(Zn)**	0.63	11.19	1.79	9.61	536.87	5.82	44.96
**I_geo_(As)**	−3.12	1.74	−0.64	0.77	120.31	0.29	0.14
**I_geo_(Cd)**	−1.8	3.37	0.47	0.75	159.57	0.98	1.77
**I_geo_(Cr)**	−2.86	3.19	−0.07	0.45	642.86	−0.93	15.29
**I_geo_(Cu)**	−2.47	4.33	−0.29	0.99	341.38	1.54	2.80
**I_geo_(Hg)**	−1.82	2.74	−0.37	0.52	140.54	1.38	6.84
**I_geo_(Pb)**	−1.60	2.34	−0.19	0.58	305.26	1.76	4.15
**I_geo_(Zn)**	−1.92	2.95	−0.36	0.55	152.78	1.10	5.69
**PI(As)**	0.17	5.01	1.12	0.69	61.61	2.05	5.78
**PI(Cd)**	0.43	15.50	2.45	1.85	75.51	3.55	16.21
**PI(Cr)**	0.21	13.73	1.50	0.71	47.33	12.87	221.56
**PI(Cu)**	0.27	30.24	2.57	3.33	129.57	4.13	20.75
**PI(Hg)**	0.43	10.00	1.26	0.78	61.90	7.68	77.99
**PI(Pb)**	0.49	7.60	1.46	0.93	63.70	3.85	17.82
**PI(Zn)**	0.39	11.58	1.28	0.81	63.28	7.30	76.34
**IPI(Ave)**	0.66	7.54	1.66	0.83	0.50	2.68	9.78

**Figure 2:**
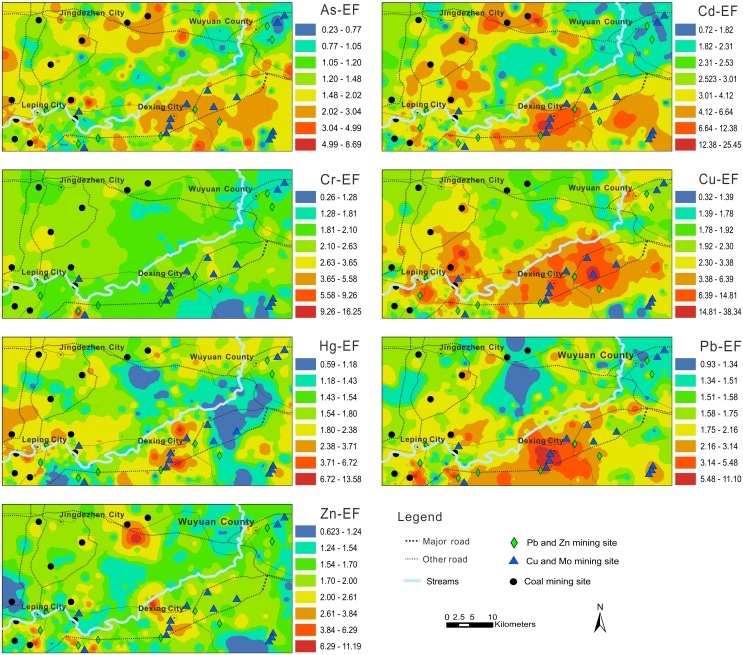
Spatial distribution of EF of trace elements relative to Jiangxi background in soil of the study area.

Estimated maps of EF of seven heavy metals in soil are presented in Fig. 2. The EF map of Cu shows higher values in areas surrounding the Dexing and Leping mining areas, which contain many Cu and Mo mining sites. The highest levels of Cd and Pb occurred at the centre of Dexing. Urban vehicular emissions and industrial activity, including incinerator operation and metallurgic activities, have continuously contributed to Cd and Pb contamination of topsoil in this area. The spatial distribution of As was highly heterogeneous in contrast to the other metals, suggesting that As in these samples may originate from point source pollution. In contrast to other heavy metals, the spatial distribution of Cr shows no clear hotspots, suggesting the study area is weakly polluted by Cr.

### Soil contamination assessment based on I_geo_

**Figure 3:**
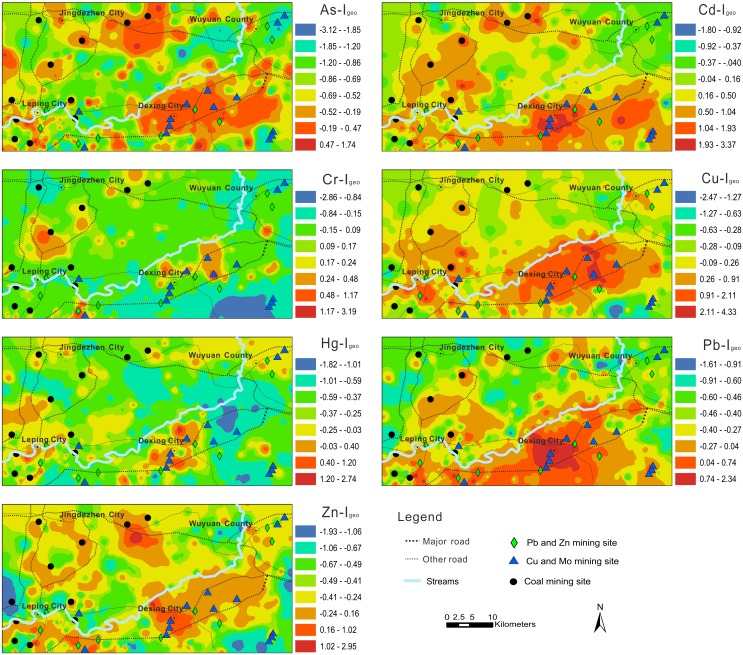
Spatial distribution of I_geo_ of trace elements in soil of the study area.

The mean I_geo_ values for all trace elements were lower than 0 (ranged from −0.07 to −0.64), suggesting a lack of soil contamination, except for Cd (Table 6). The spatial distributions of Cd, Cu, and Pb exhibited similar patterns (Fig. 3), however, the I_geo_ values indicated that the area polluted by Cd and Cu was more extensive than the area polluted by Pb. The spatial distribution of I_geo_ for Cr was similar to the EF for Cr, confirming the lack of Cr contamination. Most soils in the world do not contain elevated concentrations of Hg, which is leached and evaporates after being reduced to the metallic form, although a portion is absorbed by organic matter and clay minerals. The urban areas, including Jingdezhen, Leping, Dexing, and Wuyaun, had the highest Hg I_geo_ values; the remaining area is weakly enriched in Hg.

### Soil contamination assessment based on PI

**Figure 4:**
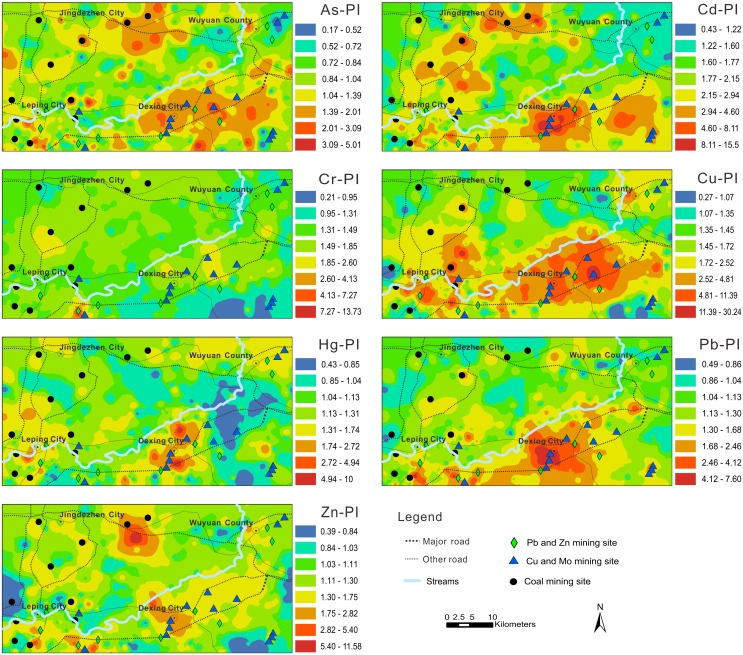
Spatial distribution of PI of trace elements in soil of the study area.

The mean PIs for all trace elements ranged from 1.12 to 2.57, which indicates that the soils were moderately contaminated (Table 6). The assessment of the overall contamination of soil was based on IPI_Ave_. The IPI_Ave_, calculated according to the mean of the PIs of the seven trace elements, was 1.66, which indicates moderate contamination. Estimated PI maps of seven heavy metals in soil are presented in Fig. 4. Among these soil contamination indices, the spatial distributions of I_geo_ and PI are remarkably similar across the study area.

### Source identification based on PCA

PCA has been extensively used to identify contamination sources. The results of the PCA conducted in this study are shown in Table 7. In this study, three principal components explained 64.36% of total variance, according to the initial eigenvalues (eigenvalues>1). As, Zn, Cd, Cu, Pb, and Hg were closely associated with the first principal component (PC1), explaining 35.71% of total variance; Cr and Ti were associated with the second principal component (PC2), which explained 16.78% of total variance; and Fe_2_O_3_ and Al_2_O_3_ were associated with the third principal component (PC3), explaining 11.87% of total variance. The other seven components (eigenvalues<1) explain little of the variability in the dataset and will not be discussed further.

**Table 7:** Total variance explained and rotated component matrix (three principal components selected) for heavy metal contents.

Element	Total	% of variance	Cumulative %	PC1	PC2	PC3
As	3.57	35.713	35.713	**0.58**	0.16	0.26
Cd	1.68	16.78	52.50	**0.79**	−0.044	0.104
Cr	1.19	11.87	64.37	0.11	**0.81**	0.30
Cu	0.86	8.61	72.97	**0.61**	0.37	0.177
Hg	0.81	8.07	81.03	**0.43**	0.24	−0.42
Pb	0.58	5.81	86.84	**0.83**	−0.05	0.41
Zn	0.45	4.51	91.36	**0.71**	0.13	0.45
Fe_2_O_3_	0.36	3.62	94.98	0.23	0.54	**0.65**
Al_2_O_3_	0.28	2.75	97.73	0.15	0.07	**0.84**
Ti	0.23	2.27	100	0.04	**0.85**	−0.13

**Figure 5:**
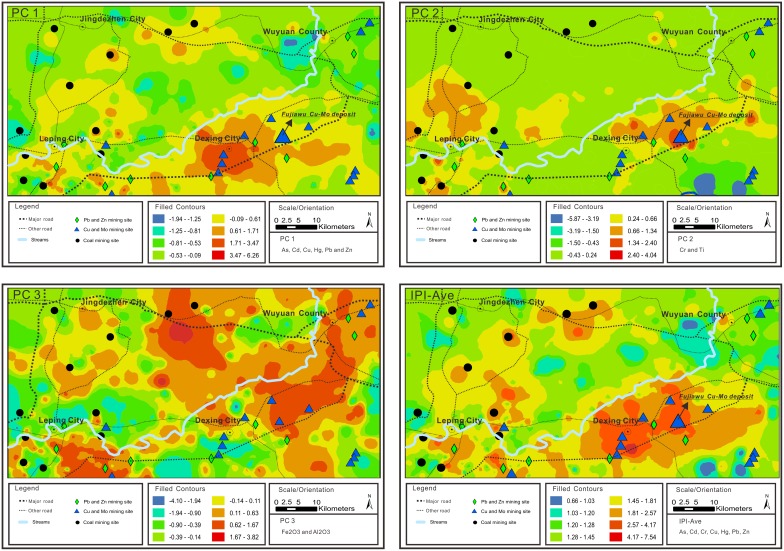
Spatial distribution map of PC scores.

As shown in this Fig. 5, high score areas were distributed in and around some of the Cu-Mo mining sites and along major roads. The areas with high component 1 scores that produced high amounts of Cd, Cu, Pb and Zn, were located around the Fujiawu Cu-Mo deposit (the biggest open store of Cu in Asia). These mining activities represented by PC1 may have be the primary contributors of Cd, Cu, Pb, and Zn contamination in soil. Thus, PC1 was mainly controlled by anthropogenic sources.

Interpolated scores associated with PC2 are displayed in Fig. 5; the scores exhibit a different spatial distribution than PC1 scores. Two high score areas were located in the city of Leping and the Fujiawu Cu-Mo deposit. The high score areas located in Leping are associated more strongly with natural sources. The reasons for the observed high scores of areas located in the Fujiawu Cu-Mo deposit are not clear; in Cu deposits, naturally occurring Cu is often present in higher concentrations than other environment. There are a number of potential causes of high PC2 scores, including the influence of anthropogenic activities. The findings suggest that Ti and Cr in soil originated from both natural and anthropogenic sources.

The spatial distribution of PC3 is presented in Fig. 5. The spatial variability of the score associated with PC3 is different than that of the scores associated with PC1 and PC2. Fe_2_O_3_ and Al_2_O_3_ were grouped into PC3, with high factor loading (Fe_2_O_3_ = 0.648; Al_2_O_3_ = 0.838). Fe_2_O_3_ and Al_2_O_3_ are ubiquitous components of soil and display some natural soil characters. It is speculated that natural sources may contribute to the Fe_2_O_3_ and Al_2_O_3_ present in the soil environment.

### Comparative method evaluation

Almost all of the four indices used in this study have been employed previous in soil contamination assessments. However, our assessments based on GIS have some distinct advantages over those done in previously studies: (1) using these maps, soil researchers and managers can visually identify the degree of anthropogenic influence on the environment at a regional scale; (2) all mapping indices incorporate some other relative information, such as land-use type, soil type, and human activities, which lead to increased confidence in the results; and (3) mapping indices can serve as a platform for planning other soil research.

Though similar integrated soil quality evaluation results were obtained from the four indices, PCA is better for than EFs, I_geo_, and PIs integrated soil contamination assessment in the study area. Using PCA, integrated soil contamination was assessed by differentiating the importance of various indicators. The 10 elements measurements constituting a dataset were included in the statistical analyses to find the influence of anthropogenic components by multivariate analysis. The drawback is that this is a qualitative method, which cannot evaluate the degree of contamination. However, IPIave treats each trace element as an independent entity and does not consider the specific geochemical context of each element.

The three soil contamination indices we used were dependent on the use of regional background values. Based on the indices, which are calculated according to mean values, Cd was classified to have caused moderate contamination, while the degree of contamination of other heavy metals varied. Using mean trace metal values/regional background ratios of soil on a regional scale is an oversimplified approach and may result in erroneous estimates of soil contamination. Thus, the use of mean values is a reliable way to evaluate contamination of an entire region because different sample material will respond differently to the presence of elements in the soil.

The mapping of the contamination indices we used, which take into account spatial information and human activities, provide an effective way to evaluate the spatial distributions of anthropogenic impact on soil composition. Using EFs, I_geo_, and PI calculated relative to off-site reference values of an entire region does not improve the sensitivity of the methods to the anthropogenic enrichment and may even give spurious results. This study demonstrates that values of contamination indices can be high relative to off-site values for a number of reasons, and contamination is just one potential cause. The three off-site references methods employed in this study are easy to conduct, and may be used for quantitative analyses to assume consistent effects of geologic and pedogenic processes at regional scale.

## Conclusions

The findings of this study suggest that EFs, I_geo_, and PI calculated according trace metal mean values relative to off-site reference values to assess soil contamination provide different interpretations of the same data. The assessment results are inconsistent, and no conclusions are reliable. However, the mapping of EFs, I_geo_, PI, and PCA, combined with contamination source analysis, has the potential to differentiate between anthropogenic and natural element sources.

The most plausible results are likely to be obtained from multivariate statistical analysis­ methods. In this study, the use of PCA allowed us to discriminate between natural and anthropogenic trace metals in soils of the study area. The results are supported by the resulting EF, I_geo_, and PI maps. According to the analysis, surface horizons are highly enriched in Cd, Cu, Pb, and Zn. The composition of topsoil is significantly modified by human activity in areas with high population density and areas near mining sites. As and Hg present in the soil were also mainly derived from anthropogenic sources, and occurred in relatively high concentrations in urban areas, in contrast to Cd, Cu, Pb, and Zn. Mapping of the soil contamination assessment indices seems to be an efficient tool for detecting sources of anomalies in the study area.

## References

[1] Andrews SS, Carroll CR (2002) Designing a soil quality assessment for sustainable agroecosystem management. Ecol Appl 11: 1573–1585.

[2] Teng YG, Wu J, Lu SJ, Wang YY, Jiao XD, et al (2014) Soil and soil environmental quality monitoring in China: A review. Environ Int 69: 177–199. 24875802 10.1016/j.envint.2014.04.014

[3] Li ZY, Ma ZW, de Tsering JVK, Yuan ZW, Huang L (2014) A review of soil heavy metal pollution from mines in China: Pollution and health risk assessment. Sci Total Environ 468–469: 843–853. 10.1016/j.scitotenv.2013.08.090 24076505

[4] Khalil A, Hanich L, Bannari A, Zouhri L, Pourret O, et al (2014) Assessment of soil contamination around an abandoned mine in a semi-arid environment using geochemistry and geostatistics: Pre-work of geochemical process modeling with numerical models. J Geochem Explor 125: 117–129.

[5] Ikem A, Campbell M, Nyirakabibi I, Garth J (2008) Baseline concentrations of trace elements in residential soils from Sourtheastern Missouri. Environ Monit Assess 140: 69–81. 17574535 10.1007/s10661-007-9848-2

[6] Desaules A (2012) Critical evaluation of soil contamination assessment methods for trace metals. Sci Total Environ 426: 120–131. 22542230 10.1016/j.scitotenv.2012.03.035

[7] Hawkes HE, Webb JS (1962) Geochemistry in Mineral Exploration. New York: Harper. 409 p.

[8] ISO: International Organisation for Standardisation (2005) Soil Quality: Vocabulary. Part 1. Terms and Definitions Relating to the Protection and Pollution of the Soil. Available: http://www.iso.org/iso/home/store/catalogue_ics/catalogue_detail_ics.htm?ics1=13&ics2=80&ics3=1&csnumber=38529.

[9] Porteous A (1996) Dictionary of Environmental Science and Technology. 2^nd^ edition. Chichester, NY: John Wiley & Sons. 794 p.

[10] Reimann C, Filzmoser P, Garrett RG (2005) Background and threshold: critical comparison of methods of determination. Sci Total Environ 346: 1–16. 15993678 10.1016/j.scitotenv.2004.11.023

[11] Garrett RG (1991) The management, analysis and display of exploration geochemical data. Exploration geochemistry workshop. Ottawa: Geological Survey of Canada. 9–1 to 9–41.

[12] Darnley AG (1995) International geochemical mapping–a review. J Geochem Explor 55: 5–10.

[13] Utermann J, Düwel O, Nagel I (2006) Contents of trace elements and organic matter in European soils. In: Gawlik BM, Bidoglio G, editors. Background values in European soils and sewage sludges. Luxembourg: European Commission. 282 p.

[14] CEMS: Chinese environmental monitoring station (1990) Background values of elements in soils of China (in Chinese). Beijing: China Environmental Press. 501 p.

[15] He J, Xu G, Zhu H, Peng G (2005) soil background values of Jiangxi Province. Beijing: Chinese Environmental Science Press. 314 p.

[16] Reimann C, de Caritat P (1998) Chemical elements in the environment-factsheets for the geochemist and environmental scientist. Berlin, Germany: Springer-Verlag. 398 p.

[17] Taylor SR, McLennan SM (1995) The geochemical evolution of the continental crust. Rev Geophys 33: 241–65.

[18] Wedepohl KH (1995) The composition of the continental Crust. Geochim Cosmochim Ac 59: 1217–32.

[19] Salminen R, Gregorauskiene V (2000) Considerations regarding the definition of a geochemical baseline of elements in the surficial materials in areas differing in basic geology. Appl Geochem 15: 647–653.

[20] CEPA: Chinese Environmental Protection Administration (1995) Environmental quality standard for soils (GB 15618-1995) (in Chinese). Available: http://kjs.mep.gov.cn/hjbhbz/bzwb/trhj/trhjzlbz/199603/W020070313485587994018.pdf.

[21] CCME: Canadian Council of Ministers of the Environment (2007) Canadian soil quality guidelines for the protection of environmental and human health. Available: http://ceqg-rcqe.ccme.ca/en/index.html.

[22] Li XD, Lee SL, Wong SC, Shi WZ, Thornton L (2004) The study of metal contamination in urban soils of Hong Kong using a GIS-based approach. Sci Total Environ 129: 113–124. 10.1016/j.envpol.2003.09.030 14749075

[23] Sepulvado JG, Blaine AC, Hundal LS, Higgins CP (2011) Occurrence and Fate of Perfluorochemicals in Soil Following the Land Application of Municipal Biosolids. Environ Sci Technol 45(19): 8106–8112. 21446724 10.1021/es103903d

[24] Nathanail CP, Earl N (2001) Human Health Risk Assessment: Guideline Values and Magic Numbers Issues in Environmental Science and Technology No.16 Assessment and Reclamation of Contaminated Land. The Royal Society of Chemistry. 85–101.

[25] Carlon C (2007) Derivation methods of soil screening values in Europe. A review and evaluation of national procedures towards harmonization. European Commission, Joint Research Center, Ispra. 206 p.

[26] Ferguson C, Darmendrail D, Freier K, Jensen BK, Jensen J, et al. (1998) Risk Assessment for Contaminated Sites in Europe. Volume 1: Scientific Basis. LQM Press, Nottingham. 165 p.

[27] Blaser P, Zimmermann S, Luster J, Shotyk W (2000) Critical examination of trace element enrichments and depletions in soils: As, Cr, Cu, Ni. Pb and Zn in Swiss forest soils. Sci Total Environ 249: 257–280. 10813458 10.1016/s0048-9697(99)00522-7

[28] Hernandez L, Probst A, Probst JL, Ulrich E (2003) Heavy metal distribution in some French forest soils: evidence for atmospheric contamination. Sci Total Environ 312: 195–219. 12873411 10.1016/S0048-9697(03)00223-7

[29] ISO: International Organisation for Standardisation (2005) Soil quality-guidance on the determination of background values. ISO 19258. Available: http://www.iso.org/iso/catalogue_detail.htm?csnumber=33772.

[30] Shotyk W, Cherkubin AK, Appleby PG, Fankhauser A, Kramers JD (1997) Lead in three peat bog profiles, Jura mountains, Switzerland: enrichment factors, isotopic composition, and chronology of atmospheric deposition. Water Air Soil Pollut 100: 297–310.

[31] Reimann C, de Caritat P (2000) Intrinsic flaws of element enrichment factors (EFs) in environmental geochemistry. Environ Sci Technol 34: 5084–5091.

[32] Hakanson L (1980) An ecological risk index for aquatic pollution control. A sedimentological approach. Water Res 14: 975–1001.

[33] Chester R, Stoner JH (1973) Pb in particulates from the lower atmosphere of the eastern Atlantic. Nature 245: 27–8.

[34] Zoller WH, Gladney ES, Duce RA (1974) Atmospheric concentrations and sources of trace metals at the Sourth Pole. Science 183: 199–201. 10.1126/science.183.4121.198 17777264

[35] Müller G (1979) Schwermetalle in den Sedimenten des Rheins-Veränderungen seit. Umschau 24: 773–8.

[36] Manta DS, Angelone M, Bellanca A, Neri R, Sprovieri M (2002) Heavy metals in urban soils: a case study from the city of Palermo (Sicily), Italy. Sci Total Environ 300: 229–243. 12685485 10.1016/s0048-9697(02)00273-5

[37] Wang XQ, He MC, Xie J, Xi JH, Lu XF (2010) Heavy metal pollution of the world largest antimony mine-affected agricultural soils in Hunan province (China). J Soil Sediment 10: 827–837.

[38] Loska K, Cebula J, Pelczar J, Wiechula D, Kwapilinski J (1997) Use of enrichment and contamination factors together with geoaccumulation indexes to evaluate the content of Cd, Cu, and Ni in the Rybnik water reservoir in Poland. Water Air Soil Pollut 93: 347–65.

[39] Loska K, Wiechula D, Korus I (2004) Metal contamination of farming soils affected by industry. Environ Int 30: 159–165. 14749104 10.1016/S0160-4120(03)00157-0

[40] Gowd SS, Reddy MR, Govil PK (2010) Assessment of heavy metal contamination in soils at Jajmau (Kanpur) and Unnao industrial areas of the Ganga Plain, Uttar Pradesh, India. J Hazard Mater 174: 113–121. 19837511 10.1016/j.jhazmat.2009.09.024

[41] USEPA: United States Environmental Protection Agency (1992) Terms of Environment. Communications Education And Public Affairs, USEPA 175-B-92-001. Available: http://iaspub.epa.gov/sor_internet/registry/termreg/searchandretrieve/termsandacronyms/search.do.

[42] Luo XS, Yu S, Zhu YG, Li XD (2012) Trace metal contamination in urban soils of China. Sci Total Environ 421–422: 17–30. 10.1016/j.scitotenv.2011.04.020 21575982

[43] Teng YG, Ni SJ, Wang JS, Niu LG (2009) Geochemical baseline of trace elements in the sediment in Dexing area, South China. Environ Geol 57: 1646–1660.

[44] Sutherland RA (1999) Distribution of organic carbon in bed sediments of Manoa Stream, Oahu, Hawaii. Earth Surf Proc Land 27: 571–583.

[45] Xie Y, Chen TB, Lei M, Yang J, Guo QJ, et al (2011) Spatial distribution of soil heavy metal pollution estimated by different interpolation methods: accuracy and uncertainty analysis. Chemosphere 82: 468–476. 20970158 10.1016/j.chemosphere.2010.09.053

[46] Teng YG, Ni SJ, Wang JS, Zuo R, Yang J (2010) A geochemical survey of trace elements in agricultural and non-agricultural topsoil in Dexing area, China. J Geochem Explor 104: 118–127.

